# Supplementary material for: Association of a CHEK2 somatic variant with tumor microenvironment calprotectin expression predicts platinum resistance in a small cohort of ovarian carcinoma
Source: PLoS One. 2025 Mar 27;20(3):e0315487. doi: 10.1371/journal.pone.0315487 (PMC11949324; doi:10.1371/journal.pone.0315487)
Supplement: S1 Table — (PDF) [file pone.0315487.s001.pdf]

S1 Table - Primary antibodies used for IHC

Antigen*	Supplier	Clone	Isotype	Specifity	Titration
**CD4+**	Cell Marque	SP35		Helper T lymphocytes	1:200
**CD8+**	Cell Marque	SP16		Cytotoxic T lymphocytes in paraffin-embedded material	1:250
**PD-L1**	Thermo Fisher	P5	PD-L1 (#PA5-20343)	Immune checkpoint	1:250
**PD-L2**	Thermo Fisher	PA5		Immune checkpoint	1:100
**L1/MAC387**	DAKO	+ MAC 373	IgG1, Kappa	M1 macrophages	1:100
**CD68+**	Abcam	ab955	IgG1	Resident macrophages	1:100

Note: * PBS was used as negative control for all IHC assays.
