# Supplementary material for: Association of a CHEK2 somatic variant with tumor microenvironment calprotectin expression predicts platinum resistance in a small cohort of ovarian carcinoma
Source: PLoS One. 2025 Mar 27;20(3):e0315487. doi: 10.1371/journal.pone.0315487 (PMC11949324; doi:10.1371/journal.pone.0315487)
Supplement: S3 Table — (PDF) [file pone.0315487.s003.pdf]

S3 Table - Demographic and clinical characteristics of patients with high-grade serous ovarian cancer (HGSOC)

**Baseline characteristics**	**n (%)**
**Mean age at diagnosis**	
< 40	01 (4.16%)
40-49	05 (20.83%)
50-59	06 (25%)
> 60	12 (50%)
**Mean parity**	
0-2	12 (50%)
3-4	06(25%)
≥ 5	04 (16.66%)
Ns	02 (8.33%)
**Menopause status**	
No	08 (33.33%)
Yes	14 (58.33%)
Ns	02 (8.33%)
**CA 125 concentration (U/mL)**	
< 35	02 (8.33%)
35-300	02 (8.33%)
301-1000	07 (29.16%)
> 1000	13 (54.16%)
**FIGO Stage**	
I - II	05 (20.83%)
III - IV	19 (79.16%)
**Peritoneal cavity-ascites**	
Yes	15 (62.51%)
No	08 (33.33%)
Ns	01 (4.16%)
**Lymphovascular space invasion**	
Yes	19 (79.16%)
No	01 (4.16%)
Ns	04 (16.66%)

FIGO: International Federation of Gynecology and Obstetrics; Ns, not specified. Values are presented as number (%).
