# Supplementary material for: Association of a CHEK2 somatic variant with tumor microenvironment calprotectin expression predicts platinum resistance in a small cohort of ovarian carcinoma
Source: PLoS One. 2025 Mar 27;20(3):e0315487. doi: 10.1371/journal.pone.0315487 (PMC11949324; doi:10.1371/journal.pone.0315487)
Supplement: S4 Table — (PDF) [file pone.0315487.s004.pdf]

S4 Table - Comparison of T cell and Macrophage levels or ratios

	Platinum-sensitivity					5 yr Overall Survival					Primary Debulking Surgery				
	PS		PR			Alive		Dead			Optimal		Suboptimal		
	Md	IQR	Md	IQR	Pv	Md	IQR	Md	IQR	Pv	Md	IQR	Md	IQR	Pv
**Cell count [1]**															
$CD4^+$	26	2-62	12	0-257	0.76	22	0-39	89	8-190	0.38	75	23-134	56	0-36	0.12
$CD8^+$	63	15-168	143	27-150	0.53	68	4-149	185	30-240	0.42	134	25-239	128	11-148	1.00
$CD68^+$	71	49-116	72	39-137	0.96	105	66-122	131	45-122	0.27	70	35-109	131	54-124	0.65
$L1^+/MAC387^+$	78	35-178	133	74-179	0.38	117	29-84	153	45-182	0.41	103	45-176	120	24-177	0.85
**Ratios [2]**															
$CD8^+/CD4^+$	2.9	0.6-4.5	6.0	2.3-21.4	0.30	4.0	1.1-6.0	2.5	0.7-8.9	0.61	1.1	0.3-12.0	4.0	0.9-8.9	0.30
$CD8^+/CD68^+$	0.7	0.1-2.5	1.9	0.4-2.3	0.59	0.3	0-2.2	1.7	0.4-4.0	0.11	1.4	0.3-6.7	0.7	0.2-2.3	0.56
$CD68^+$/L1MAC387	1.0	0.3-3.7	0.5	0.4-0.7	0.59	1.7	0.5-4.2	0.5	0.3-1.0	0.15	0.4	0.3-3.0	0.8	0.4-3.0	0.43

Note: 1- Sum of cell counts of all 20 fields. 2- Ratio of the sum of all 20 fields. IQR- interquartile range; statistically significant changes are shown in bold. PS- platinum-sensitive. PR-platinumresistant.
