# Supplementary figures and images for: Association of a CHEK2 somatic variant with tumor microenvironment calprotectin expression predicts platinum resistance in a small cohort of ovarian carcinoma
Source: PLoS One. 2025 Mar 27;20(3):e0315487. doi: 10.1371/journal.pone.0315487 (PMC11949324; doi:10.1371/journal.pone.0315487)

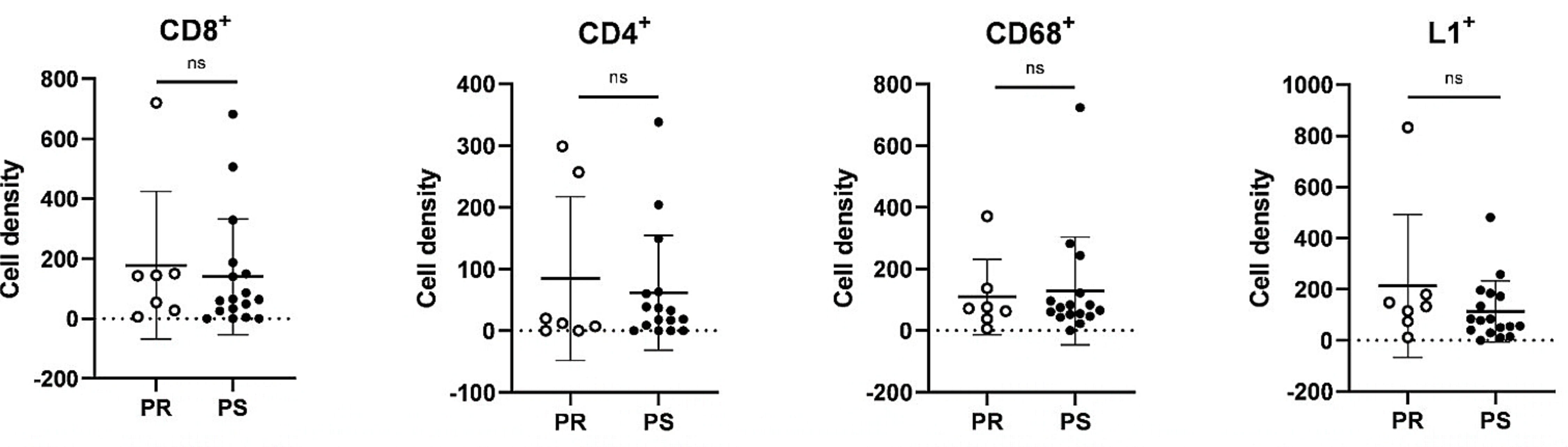

Supplement: S2 Fig — Comparison of immunological markers average expression between patients platinum sensitive (PS) and resistant (PR) was performed using Mann-Whitney test. ns = not significant. (TIF) [file pone.0315487.s008.tif]

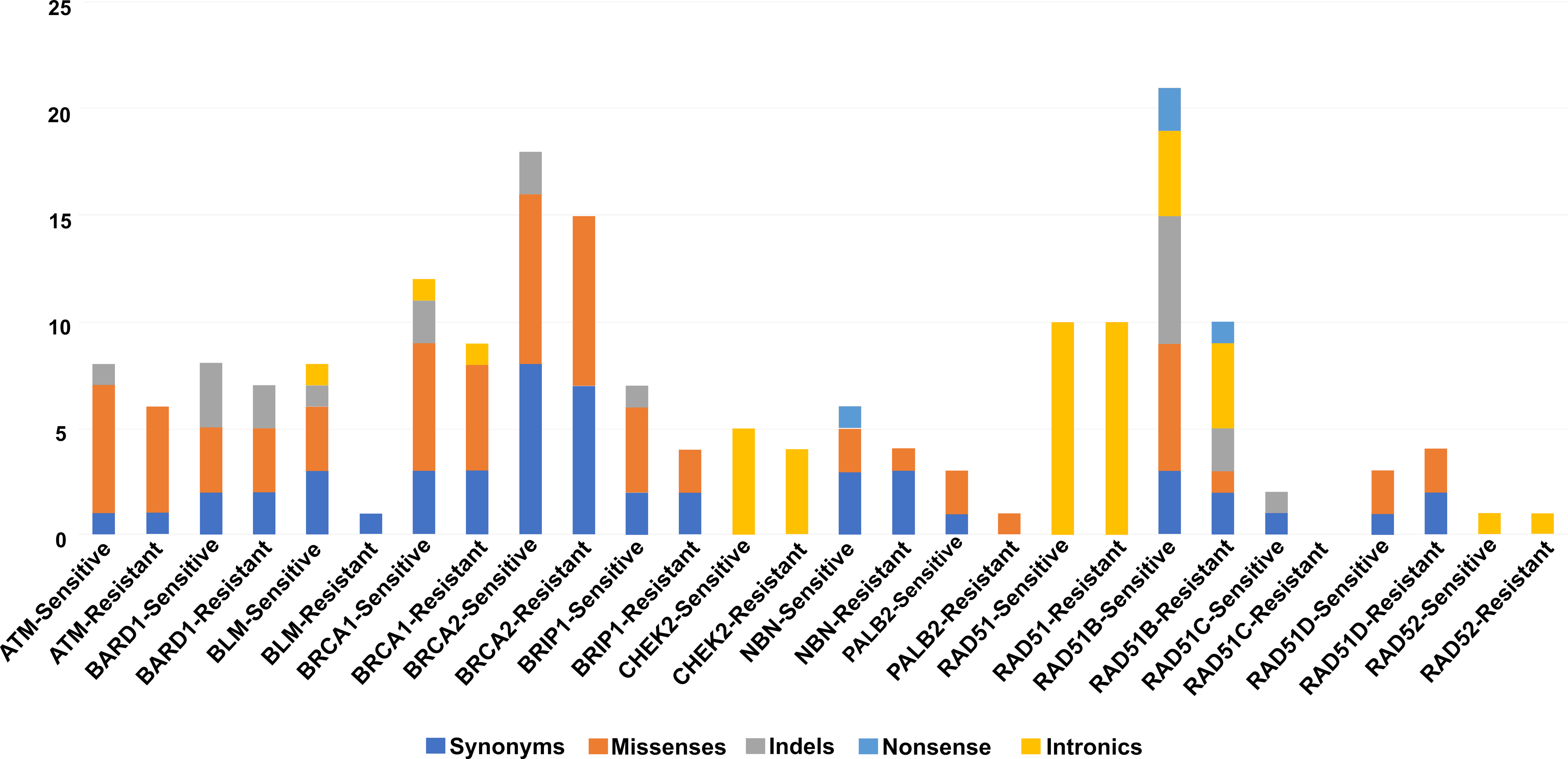

Supplement: S3 Fig — Each variant type is associated with a specific color, as illustrated. Variant types are distributed according to the genes, highlighting the predominance of intronic variants (represented in green). (TIF) [file pone.0315487.s009.tif]

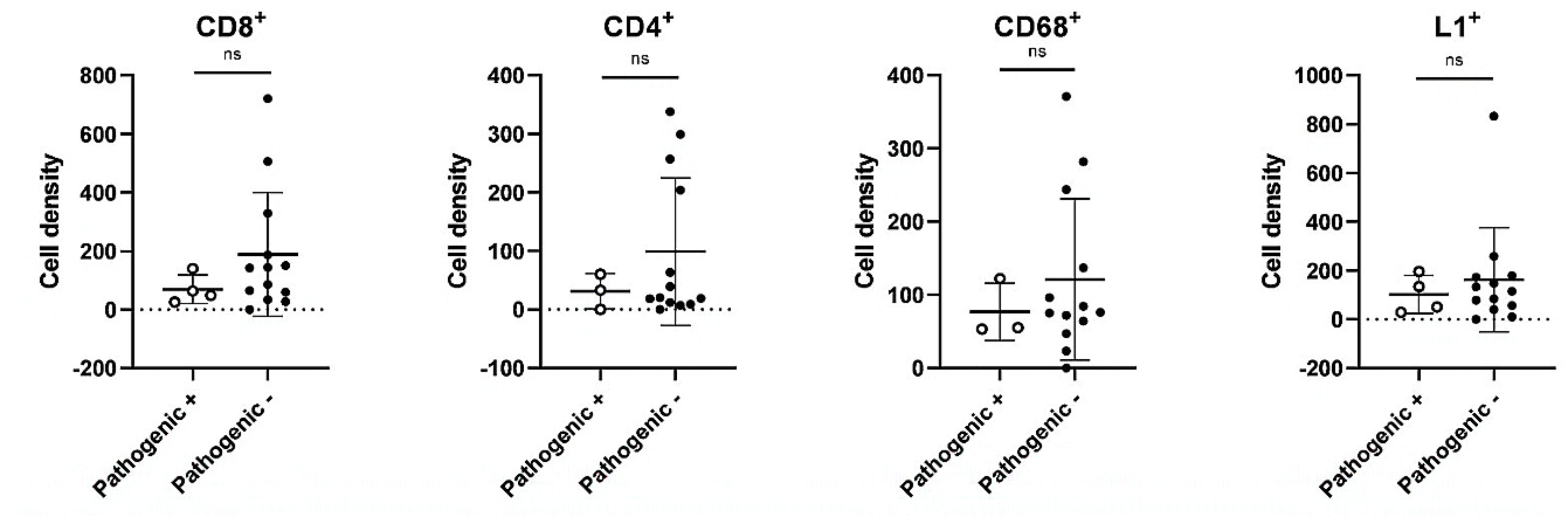

Supplement: S4 Fig — Comparison of immunological markers average expression between patients with and without pathogenic variants in any of the HR pathway genes screened by the Pan-Cancer panel was performed using Mann-Whitney test. ns = not significant. (TIF) [file pone.0315487.s010.tif]

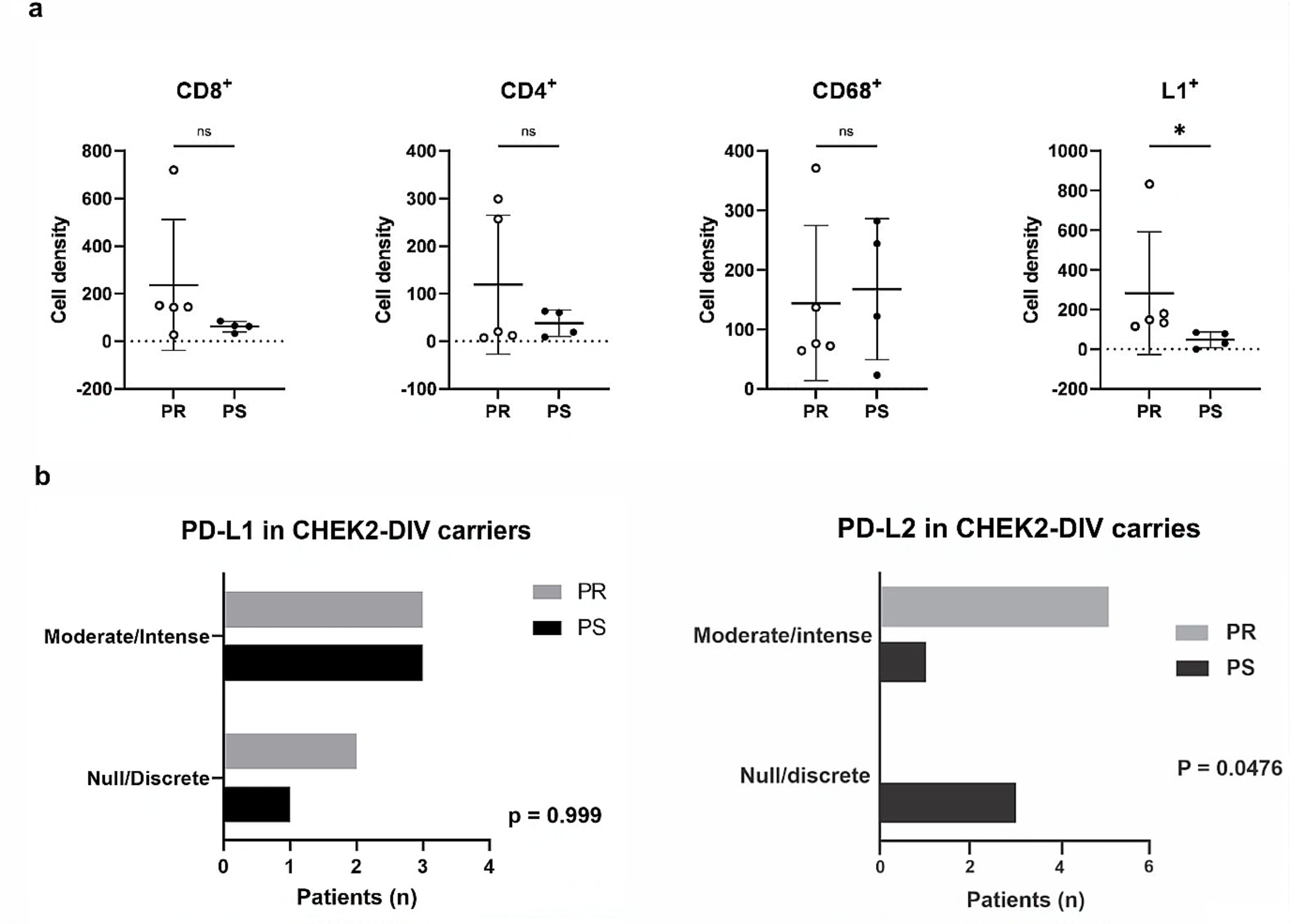

Supplement: S5 Fig — Comparison of immunological markers average expression between CHEK2-DIV patients sensitive and resistant to platinum was performed using Mann-Whitney test (a) or Fisher exact test (b). Only L1 and PD-L2 were statistically significant higher in PR patients. (TIF) [file pone.0315487.s011.tif]

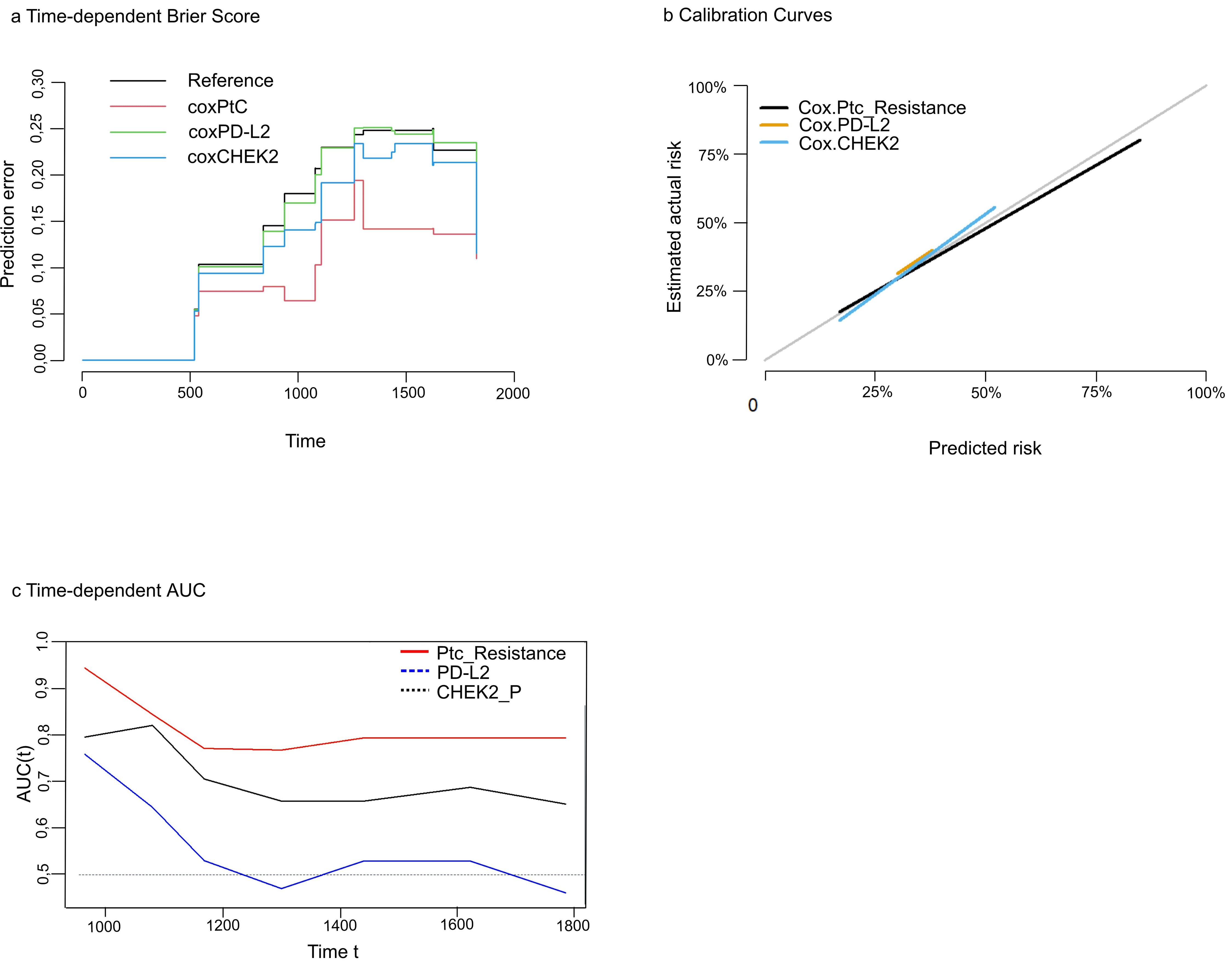

Supplement: S6 Fig — (a) Time-dependent Brier Score. The lower the Brier Score, the higher the model prediction capacity. (b) Calibration plot. Here, we observe that PD-L2 model is not well calibrated with few points lying in a straight line. On the other hand, PtC-Resistance model is very close to the diagonal line, which represents a perfect calibration. (c) The time-dependent AUC shows that PtC-Resistance and CHEK2-DIV models keep a good discriminatory power along the follow-up time, while PD-L2 model loses performance significantly after the first 2.5 years. The time range considered for this analysis corresponds to the time needed to occur 20% to 80% of events. (TIF) [file pone.0315487.s012.tif]

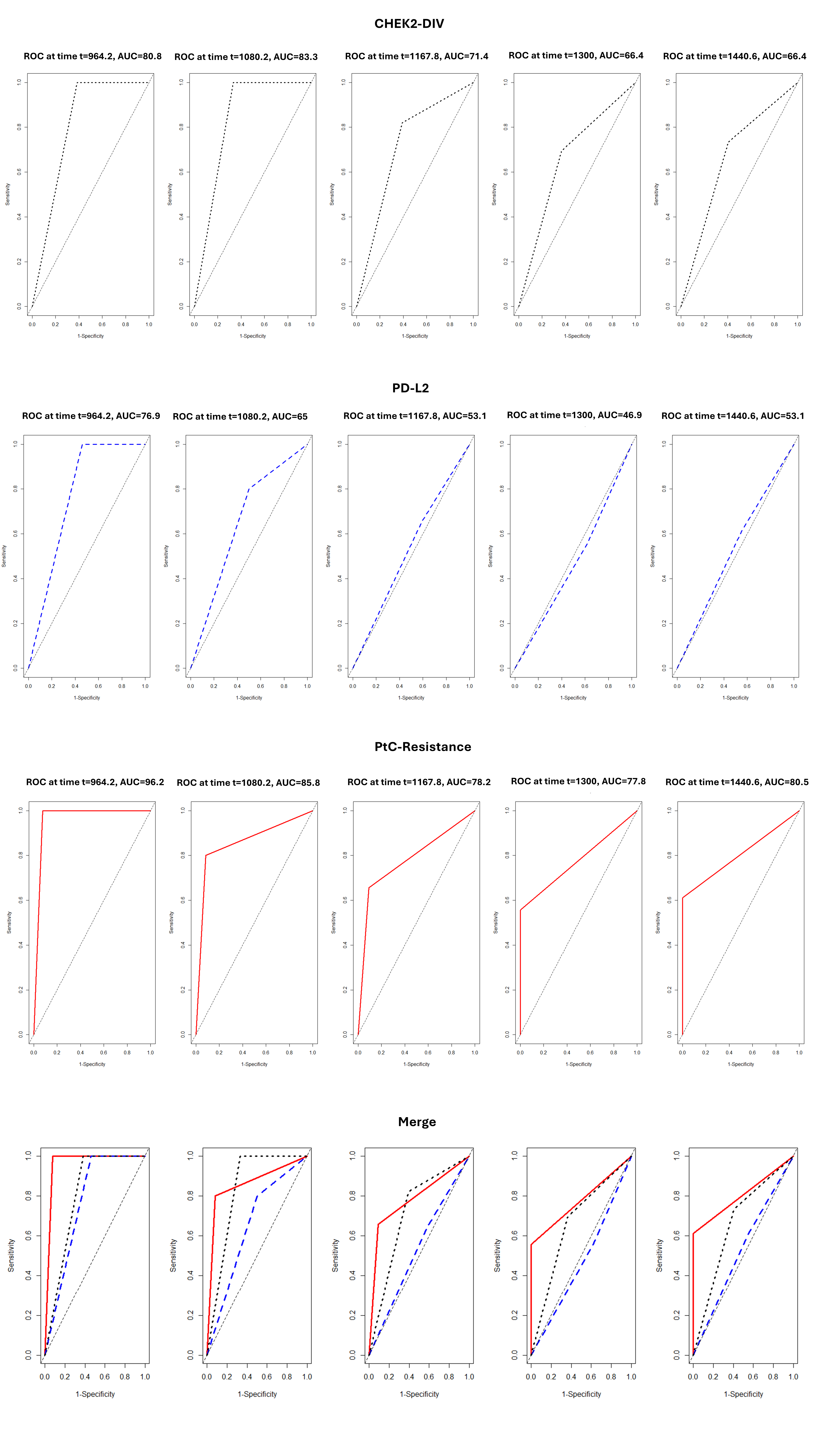

Supplement: S7 Fig — From top to bottom: CHEK2-DIV model (black line), PD-L2 model (blue line), PtC-Resistance model (red line), and the three plots merged. Here we can observe how PD-L2 model loses discriminatory power with time. (TIF) [file pone.0315487.s013.tif]
