# Supplementary material for: Association of a CHEK2 somatic variant with tumor microenvironment calprotectin expression predicts platinum resistance in a small cohort of ovarian carcinoma
Source: PLoS One. 2025 Mar 27;20(3):e0315487. doi: 10.1371/journal.pone.0315487 (PMC11949324; doi:10.1371/journal.pone.0315487)
Supplement: S1 Box — (PDF) [file pone.0315487.s014.pdf]

Box 1 - Details of the variant calling protocol

Specifically for indel variants, filters were applied to remove indels with weak evidence. Valid indels had to meet the following criteria to be included in the analysis: length equal to or greater than 20 bases, score equal to or greater than 25, and complexity equal to or greater than 13. Long indel variants with weak evidence were also removed, with only those meeting the following criteria being retained: frequency equal to or greater than 2, score equal to or greater than 50, and complexity equal to or greater than 35. The same filter was applied to long inversion variants.

Regarding low-frequency variants, those with a significance level less than 1.0% were disregarded. Additionally, filters related to the quality of the sequenced reads, coverage (Minimum coverage - 10 or more reads), read length (Minimum read length - 20 bases or more), the minimum number of reads containing the variant (Minimum count - 2 or more reads), and the minimum frequency of the variant in relation to the total number of sequenced reads (Minimum frequency - 0.5%) were applied.
