# Supplementary material for: Association of a CHEK2 somatic variant with tumor microenvironment calprotectin expression predicts platinum resistance in a small cohort of ovarian carcinoma
Source: PLoS One. 2025 Mar 27;20(3):e0315487. doi: 10.1371/journal.pone.0315487 (PMC11949324; doi:10.1371/journal.pone.0315487)
Supplement: S2 Box — (PDF) [file pone.0315487.s015.pdf]

Code for the analysis performed using RStudio.

```
#Univariate Cox Regression

library("survival")

library("survminer")


#To apply the univariate coxph function to multiple covariates at once:

covariates <- c("PDL1", "PDL2", "CD8", "CD4", "CD68","L1", "PtC_Resistance","CHEK2_P", "Age")

univ_formulas <- sapply(covariates, function(x) as.formula(paste('Surv(Time_5yr, Status_5yr)~', x)))

univ_models <- lapply( univ_formulas, function(x){coxph(x, data = Cox_3)})


# Extract data

univ_results <- lapply(univ_models,

                       function(x){

                         x <- summary(x)

                         p.value<-signif(x$wald["pvalue"], digits=2)

                         wald.test<-signif(x$wald["test"], digits=2)

                         beta<-signif(x$coef[1], digits=2);#coeficient beta

                         HR <-signif(x$coef[2], digits=2);#exp(beta)

                         HR.confint.lower <- signif(x$conf.int[,"lower .95"], 2)

                         HR.confint.upper <- signif(x$conf.int[,"upper .95"],2)

                         HR <- paste0(HR, " (",  HR.confint.lower, "-", HR.confint.upper, ")")

                         res<-c(beta, HR, wald.test, p.value)

                         names(res)<-c("beta", "HR (95% CI for HR)", "wald.test", "p.value")

                         return(res) #return(exp(cbind(coef(x),confint(x))))

                       })

res <- t(as.data.frame(univ_results, check.names = FALSE))

as.data.frame(res)

###########################################################################################
```

```
#Adjusted survival curves for Cox model

library(survival)

Cox_3<-as.data.frame(Cox_3)

#changing PDL2 to Null/Discrete x Moderate/Intense

Cox_3$PDL2<-ifelse(Cox_3$PDL2==1,0,Cox_3$PDL2)

Cox_3$PDL2<-ifelse(Cox_3$PDL2==2,1,Cox_3$PDL2)

Cox_3$PDL2<-ifelse(Cox_3$PDL2==3,1,Cox_3$PDL2)


fit.PtC <- coxph( Surv(Time_5yr, Status_5yr) ~ PtC_Resistance, data = Cox_3 )

# average in groups

ggadjustedcurves(fit.PtC, data = Cox_3, method = "average", variable = "PtC_Resistance", legend.title =
"PtC_Resistance")

curve.PtC <- surv_adjustedcurves(fit.PtC, data = Cox_3, method = "average", variable = "PtC_Resistance")


fit.CHEK2 <- coxph( Surv(Time_5yr, Status_5yr) ~ CHEK2_P, data = Cox_3 )

# average in groups

ggadjustedcurves(fit.CHEK2, data = Cox_3, method = "average", variable = "CHEK2_P", legend.title = "CHEK2")

curve.CHEK2 <- surv_adjustedcurves(fit.CHEK2, data = Cox_3, method = "average", variable = "CHEK2_P")


fit.PDL2 <- coxph( Surv(Time_5yr, Status_5yr) ~ PDL2, data = Cox_3 )

# average in groups

ggadjustedcurves(fit.PDL2, data = Cox_3, method = "average", variable = "PDL2", legend.title = "PDL2")

curve.PDL2 <- surv_adjustedcurves(fit.PDL2, data = Cox_3, method = "average", variable = "PDL2")


###############################################################################################
```

```
# Computing time-dependent Brier Score

library(pec)

library(prodlim)

library(survival)

Cox.subset<-na.omit(subset(Cox_3, select=-c(PDL1, CD4, CD8, CD68, L1, Age))) #removing variables that will not be used and then NA values

Models <- list("coxPtC"=coxph(Surv(Time_5yr,Status_5yr)~PtC_Resistance,data=Cox.subset,x=TRUE,y=TRUE),

           "coxPDL2"=coxph(Surv(Time_5yr,Status_5yr)~PDL2,data=Cox.subset,x=TRUE,y=TRUE),

           "coxCHEK2"=coxph(Surv(Time_5yr,Status_5yr)~CHEK2_P,data=Cox.subset,x=TRUE,y=TRUE))


# Computing the apparent prediction error (weighted mean of the Brier score over time)

PredError <- pec(object=Models,

                 formula=Surv(Time_5yr,Status_5yr)~PtC_Resistance+PDL2+CHEK2_P,

                 data=Cox.subset,

                 exact=TRUE,

                 cens.model="cox",

                 splitMethod="none",

                 B=0,

                 verbose=TRUE)

print(PredError,times=seq(1,5,1)*365)

summary(PredError)

plot(PredError,xlim=c(0,6)*365)

###########################################################################################

#Ploting Calibration Curve

library(riskRegression)


xs=Score(list("Cox.PtC_Resistance"=coxPtC,"Cox.PDL2"=coxPDL2,"Cox.CHEK2"=coxCHEK2),

          Surv(Time_5yr,Status_5yr)~1,data=Cox.subset, plots="cal",metrics="brier", split.method= "none")

plotCalibration(xs, method="nne", brier.in.legend = TRUE)

###########################################################################################
```

```
#Computing time-dependent ROC and AUC
library(timeROC)
ROC.PtC_Resistance<-timeROC(T=Cox.subset$Time_5yr,
                  delta=Cox.subset$Status_5yr,marker=Cox.subset$PtC_Resistance,
                  cause=1,weighting="cox",
                  times=quantile(Cox.subset$Time_5yr,probs=seq(0.2,0.8,0.1)),
                  iid=FALSE)
ROC.PDL2<-timeROC(T=Cox.subset$Time_5yr,
            delta=Cox.subset$Status_5yr,marker=Cox.subset$PDL2,
            cause=1,weighting="cox",
            times=quantile(Cox.subset$Time_5yr,probs=seq(0.2,0.8,0.1)),
            iid=FALSE)
ROC.CHEK2_P<-timeROC(T=Cox.subset$Time_5yr,
             delta=Cox.subset$Status_5yr,marker=Cox.subset$CHEK2_P,
             cause=1,weighting="cox",
             times=quantile(Cox.subset$Time_5yr,probs=seq(0.2,0.8,0.1)),
             iid=FALSE)
ROC.PtC_Resistance #print results for PtC_Resistance
ROC.PDL2 #print results for PDL2
ROC.CHEK2_P #print results for CHEK2_P

#Plot function for time-dependent ROC curve
layout(matrix(1:5, byrow = T, ncol = 5))
times=quantile(Cox.subset$Time_5yr,probs=seq(0.2,0.8,0.1))
times_plot<-as.vector(times)
for (i in 1:5){
  plot(ROC.PtC_Resistance,time=times_plot[i],lwd=2,title=TRUE)
  plot(ROC.PDL2,time=times_plot[i],col="blue",add=TRUE,lwd=2,lty=2)
  plot(ROC.CHEK2_P,time=times_plot[i],col="black",add=TRUE,lwd=2,lty=3)
}
```

```
#Plot ROC curves separately
layout(matrix(1:5, byrow = T, ncol = 5))
for (i in 1:5){
  plot(ROC.PtC_Resistance,time=times_plot[i],lwd=2,title=TRUE)
}

layout(matrix(1:5, byrow = T, ncol = 5))
for (i in 1:5){
  plot(ROC.PDL2,time=times_plot[i],col="blue",lwd=2,lty=2, title=TRUE)
}

layout(matrix(1:5, byrow = T, ncol = 5))
for (i in 1:5){
  plot(ROC.CHEK2_P,time=times_plot[i],col="black",lwd=2,lty=3, title=TRUE)
}

# Plot time-dependent AUC curve
layout(matrix(1, byrow = T, ncol = 1))
plotAUCcurve(ROC.PtC_Resistance, add = FALSE, col = "red")
plotAUCcurve(ROC.PDL2, add = TRUE, col = "blue")
plotAUCcurve(ROC.CHEK2_P, add = TRUE, col = "black")
# add legend
legend("topright",c("PtC_Resistance","PDL2","CHEK2_P"),
      col=c("red","blue","black"),lty=1:3)


##################################################################
```
