# Supplementary material for: Association of a CHEK2 somatic variant with tumor microenvironment calprotectin expression predicts platinum resistance in a small cohort of ovarian carcinoma
Source: PLoS One. 2025 Mar 27;20(3):e0315487. doi: 10.1371/journal.pone.0315487 (PMC11949324; doi:10.1371/journal.pone.0315487)
Supplement: Attachment — Submitted filename: Response to reviewers.pdf [file pone.0315487.s018.pdf]

RESPONSE TO REVIEWERS

Article title: Association of a *CHEK2* Somatic Variant with Tumor Microenvironment Calprotectin Expression Predicts Platinum Resistance in a Small Cohort of Ovarian Carcinoma

Authors: Izabela Ferreira Gontijo de Amorim, Carolina Pereira de Souza Melo, Ramon de Alencar Pereira, Sidnéa Macioci Cunha, Thalía Rodrigues de Souza Zózimo, Fábio Ribeiro Queiroz, Iago de Oliveira Peixoto, Luciana Maria Silva Lopes, Laurence Rodrigues do Amaral, Matheus de Souza Gomes, Juliana Almeida Oliveira, Eduardo Batista Cândido, Paulo Guilherme de Oliveira Salles, Letícia da Conceição Braga

Date: October 30, 2024

Submission Number: PONE-D-24-16271

We would like to thank the reviewers for their comments on our manuscript. We believe that these contributions will be important in helping us to build a clearer and higher-quality manuscript for the global scientific community. In response to your specific comments, we have made the following revisions:

Journal Requirements

Please review your reference list to ensure that it is complete and correct. If you have cited papers that have been retracted, please include the rationale for doing so in the manuscript text or remove these references and replace them with relevant current references. Any changes to the reference list should be mentioned in the rebuttal letter that accompanies your revised manuscript. If you need to cite a retracted article, indicate the article's retracted status in the References list and also include a citation and full reference for the retraction notice

Thank you for raising this important question.

- In line 138, which refers to the protocol used for the immunohistochemistry technique, the authors' names of the article that were used as the reference were previously written. We removed the name and left only the numerical citation.
- In lines 490 to 493, we reordered the citations to match the sequence they are mentioned in the manuscript text.

- We have reviewed all citations in the references section and adjusted them to Vancouver style.

Review Comments to the Author

Reviewer #3

Reviewer #3: The manuscript is interesting and covers the contemporary problem of predicting chemo-resistance in HGSOC. The main weakness of the study is its low patients number. My main point of interest is how did Authors choose the tumors for the study. I realize that all of them were HGSOC, but there are several subtypes according to the immunoreactivity of the host against tumor. The immunological composition of all the tumors studied seems to be quite uniform in its TIL's pattern. Could Authors explain this?

Thank you for your suggestion. This is a retrospective study that initially reviewed the medical records of 52 patients diagnosed with ovarian cancer treated at the Mário Penna Institute between 2015 and 2019. Of these, 24 patients were included in the study for meeting the inclusion criteria: confirmed diagnosis of high-grade serous carcinoma (HGSOC) with a pathological report, as well as complete clinical and follow-up data. The remaining 28 patients were excluded for the following reasons: 16 had histology different from HGSOC; 8 underwent surgery at another institution, resulting in loss of follow-up; and 4 had inadequate biological material (slides and paraffin blocks) for the necessary processing. To provide a clear understanding of the criteria applied, we included a flowchart in the Supplementary Files (S1_Fig), detailing the selection process for the patients with HGSOC included in this study. To clarify this modification, we added the following text in the "Patients and tissue samples" section – lines "117-120" of the Materials and Methods: "For this, the medical records of 52 patients diagnosed with ovarian cancer between 2015 and 2019 were initially reviewed. After applying the inclusion and exclusion criteria, 24 patients with high-grade serous carcinoma were selected"

Despite the apparent uniformity in the pattern of TILs, patients exhibited different distribution and quantification patterns. For instance, some showed a diffuse profile with a higher infiltration rate compared to others. However, due to the small sample size, it was not possible to identify or classify a significant difference between the groups or patients. This is evident in the results presented in graph (S2_Fig). We acknowledge that the sample size is relatively small, limiting the generalizability of our findings. However,

with an increased sample size, the apparent uniformity of the infiltration may not be sustained. Besides that, exploratory studies are essential for generating hypotheses. Additional studies with a larger number of patients and using other molecular biology techniques may provide more robust validation of these results. It is also important to consider the significant presence of several variants, including indels, intronic, missense and nonsense. In the context of cancer genomes, they can lead to altered or truncated proteins. When these altered proteins are processed by the proteasome, and the peptide fragments are presented by HLA molecules on the surface of tumor cells, these peptides can act as neoantigens, making them potential targets for the immune response. Although the presence of neoantigens may explain the occurrence of TILs in the majority of tissue samples, it is important to note that TIL analysis alone is insufficient to demonstrate the immunoheterogeneity of HGSOC. To address these limitations more clearly and highlight the study's significant contributions, we have revised the 'Conclusion section'. We now explicitly state, "Despite the study's limitations, such as the small sample size, its unicentric design, and convenience sampling, its primary strength lies in its potential for improved stratification of ovarian cancer patients regarding Pt-C resistance. Additional studies with a more significant number of patients could improve and validate these results."

We hope we have addressed correctly all the requirements and questions.

Kind regards,

Letícia Braga
