# Supplementary material for: Association of a CHEK2 somatic variant with tumor microenvironment calprotectin expression predicts platinum resistance in a small cohort of ovarian carcinoma
Source: PLoS One. 2025 Mar 27;20(3):e0315487. doi: 10.1371/journal.pone.0315487 (PMC11949324; doi:10.1371/journal.pone.0315487)
Supplement: Attachment — Submitted filename: Response to reviewers.docx [file pone.0315487.s017.docx]

**Response to Reviewers**

**Article title:** Association of a *CHEK2* Somatic Variant with Tumor Microenvironment Calprotectin Expression Predicts Platinum Resistance in a Small Cohort of Ovarian Carcinoma

**Authors:** Izabela Ferreira Gontijo de Amorim, Carolina Pereira de Souza Melo^,^ Ramon de Alencar Pereira, Sidnéa Macioci Cunha, Thalía Rodrigues de Souza Zózimo, Fábio Ribeiro Queiroz, Iago de Oliveira Peixoto, Luciana Maria Silva Lopes, Laurence Rodrigues do Amaral, Matheus de Souza Gomes, Juliana Almeida Oliveira, Eduardo Batista Cândido, Paulo Guilherme de Oliveira Salles, Letícia da Conceição Braga

**Date: September 5, 2024**

**Submission Number**: PONE-D-24-16271

We would like to express our gratitude to the reviewers for their detailed and constructive comments on our manuscript. Your observations have significantly improved the quality of our work. We have comprehensively revised the manuscript to enhance the clarity and accuracy of our descriptions. In response to your specific comments, we have made the following revisions:

**Journal Requirements**

1. Please ensure that your manuscript meets PLOS ONE's style requirements, including those for file naming. The PLOS ONE style templates can be found at 
https://journals.plos.org/plosone/s/file?id=wjVg/PLOSOne_formatting_sample_main_body.pdf and 
https://journals.plos.org/plosone/s/file?id=ba62/PLOSOne_formatting_sample_title_authors_affiliations.pdf

**Answer 1:** All file names have been revised to meet PLOS ONE’s requirements.

2. Please ensure that you include a title page within your main document. We do appreciate that you have a title page document uploaded as a separate file, however, as per our author guidelines (http://journals.plos.org/plosone/s/submission-guidelines#loc-title-page) we do require this to be part of the manuscript file itself and not uploaded separately.
Could you therefore please include the title page into the beginning of your manuscript file itself, listing all authors and affiliations.

**Answer 2:** We are sorry for the inconvenience. We have now included the title page in into the beginning of the manuscript file.

3. Please ensure that you include a title page within your main document. You should list all authors and all affiliations as per our author instructions and clearly indicate the corresponding author.

**Answer 3:** We are sorry for the inconvenience. We have now included the title page in into the beginning of the manuscript file.

**Review Comments to the Author**

**Reviewer #1**

**Comment 1:** It is stated: Yes - all data are fully available without restrictions. Unfortunately, the data were not included in the submission. Please provide the raw data, either as supplementary CSV files or via a repository, such as Mendeley Data.

**Answer 1:** We apologize for this inconvenience. We have taken your comment seriously and have thoroughly revised all tables containing clinical data and other relevant datasets. These are now being submitted along with the manuscript as S2 Table, ensuring the integrity and transparency of our research. During the revision process, we found a mistake in two event/censored patients. The correction resulted in a slight change in hazard ratio values, which have been adjusted in the manuscript. The topic is Survival Analysis of the Results session.

**Comment 2:** The authors performed LOOCV to assess the performance of the two selected predictors. However, they failed to incorporate the feature selection itself into the cross-validation framework. Consequently, the findings may be misleading.

**Answer 2:** Thank you for your insightful comment. We understand the importance of embedding feature selection within the cross-validation framework to avoid potential bias. However, we would like to clarify that the selection of the two predictors was performed independently before the application of the LOOCV, and these predictors were fixed throughout the entire validation process. This means that the feature selection was not part of the LOOCV process itself but was conducted as a preliminary step. The LOOCV was then applied to evaluate the performance of the classifiers using these pre-selected predictors. Therefore, there was no need to include the feature selection within each fold of the LOOCV, as the attributes used in the model were already determined in advance. This approach ensures that the model's performance is assessed based on the chosen predictors without any additional feature selection bias during the validation phase. We hope this clarifies our methodology and are open to further discussion if necessary.

**Comment 3:** Please complement the survival analysis already performed by the predictive approach: Use the time-dependent Brier score to quantify how well the model can predict survival. Use time-dependent ROC to quantify how well the model can discriminate. Check whether the survival predictions are well-calibrated.

**Answer 3:** Thank you for pointing out these complementary analyses. They are now presented in Supplementary Files (S6 Table, and S5 and S6 Figures). A brief citation was also included in the Statistical Analysis topic of the Material and Methods session and in the Survival Analysis topic of the Results session.

**Comment 4:** Please use the survival plots adjusted based on the adjusted CoxPH model, instead of the Kaplan-Meier plots.

**Answer 4:** As recommended, the Kaplan-Meier plots (Figure 5 b-d) were substituted by correspondent adjusted survival curves for the CoxPH Model. A correction was also made in the Statistical Analysis topic of the Material and Methods session.

**Reviewer #2**

**Comment 1a:** Lines 229-238. The authors used a decision tree classifier to identify variables and cutoffs that discriminate between the two classes PR and PS. The classifier successfully discriminates between the two classes when using all classes to fit the model. I believe this model is the one that produces the cutoff of 84 for L1 levels. I think this should be mentioned.

**Answer 1a:** Thank you for your suggestion. This information was inserted in the Results session, line 286.

**Comment 1b:** For leave one out cross validation (LOOCV). How did you choose which sample to leave out? Did you fit all 17 possible classification models? If so, is the 14/17 accuracy the best or worst accuracy among all models?

**Answer 1b:** We appreciate your question regarding the LOOCV process. In the LOOCV approach, each of the 17 samples was systematically left out once, and the model was trained on the remaining 16 samples. This process was repeated 17 times, leaving out a different sample each time. The accuracy of 14/17 reflects the number of correct predictions out of the 17 folds. To clarify, this accuracy is calculated by averaging the performance across all folds rather than representing the performance of a single model. Therefore, it reflects the overall generalizability of the model across the entire dataset rather than being the best or worst model accuracy. We will ensure this explanation is more clearly articulated in the revised manuscript.

**Comment 1c:** I suggest the authors use the term “decision tree classifier” instead of just “machine learning”.

**Answer 1c:** Thank you for your suggestion. The text became clearer and more focused with this change. The revisions have been made on lines 49, 285, and 315.

**Comment 2a:** Data and code availability. The authors performed the analysis using RStudio. It would be helpful if the corresponding scripts were included in the submission as supporting information, along with the files needed to run the code.

**Answer 2a:** All scripts used in RStudio are described in Supplementary Methods Box 2.

**Comment 3:** There is a typo in the title; the gene name should be CHEK2 instead of CHECK2. Line 168, duplicate word “Table” and line 191, and please choose a way to spell PDL1 or PD-L1.

**Answer 3:** Thank you for your careful review of our writing. All suggestions have been accepted and successfully corrected. You can track the changes in the following lines:

- a. Corrected the gene name in the title.
- b. Deleted one of the duplicate words, "Table," on line 212.
- c. We apologize for the inconsistency in referring to the biomarker. The correct form is PD-L1, which has been corrected throughout the text.

We hope we have addressed correctly all the requirements and questions.

Kind regards,

Letícia Braga
